# The Prognostic Potential of Insulin-like Growth Factor-Binding Protein 1 for Cardiovascular Complications in Peripheral Artery Disease

**DOI:** 10.3390/jcdd12070253

**Published:** 2025-07-01

**Authors:** Ben Li, Farah Shaikh, Houssam Younes, Batool Abuhalimeh, Abdelrahman Zamzam, Rawand Abdin, Mohammad Qadura

**Affiliations:** 1 Department of Surgery, University of Toronto, Toronto, ON M5S 1A1, Canada; benx.li@mail.utoronto.ca; 2 Division of Vascular Surgery, St. Michael’s Hospital, Unity Health Toronto, University of Toronto, Toronto, ON M5B 1W8, Canada; 3 Institute of Medical Science, University of Toronto, Toronto, ON M5S 1A1, Canada; 4 Temerty Centre for Artificial Intelligence Research and Education in Medicine (T-CAIREM), University of Toronto, Toronto, ON M5S 1A1, Canada; 5 Heart, Vascular, & Thoracic Institute, Cleveland Clinic Abu Dhabi, Abu Dhabi 112412, United Arab Emirates; 6 Department of Medicine, McMaster University, Hamilton, ON L8S 4L8, Canada; 7 Li Ka Shing Knowledge Institute, St. Michael’s Hospital, Unity Health Toronto, University of Toronto, Toronto, ON M5B 1W8, Canada

**Keywords:** growth factors, insulin-like growth factor-binding protein 1, major adverse cardiovascular events, prognosis, peripheral artery disease

## Abstract

Background/Objectives: Patients with peripheral artery disease (PAD) have a heightened risk of major adverse cardiovascular events (MACE), including myocardial infarction, stroke, and death. Despite this, limited progress has been made in identifying reliable biomarkers to prognosticate such outcomes. Circulating growth factors, known to influence endothelial function and the progression of atherosclerosis, may hold prognostic value in this context. The objective of this research was to evaluate a broad range of blood-based growth factors to investigate their potential as predictors of MACE in patients diagnosed with PAD. Methods: A total of 465 patients with PAD were enrolled in a prospective cohort study. Baseline plasma levels of five different growth factors were measured, and participants were monitored over a two-year period. The primary outcome was the occurrence of MACE within those two years. Comparative analysis of protein levels between patients who did and did not experience MACE was performed using the Mann–Whitney U test. To assess the individual prognostic significance of each protein for predicting MACE within two years, Cox proportional hazards regression was performed, adjusting for clinical and demographic factors including a history of coronary and cerebrovascular disease. Subgroup analysis was performed to assess the prognostic value of these proteins in females, who may be at higher risk of PAD-related adverse events. Net reclassification improvement (NRI), integrated discrimination improvement (IDI), and area under the receiver operating characteristic curve (AUROC) were calculated to assess the added value of significant biomarkers to model performance for predicting 2-year MACE when compared to using demographic/clinical features alone. Kaplan–Meier curves stratified by IGFBP-1 tertiles compared using log-rank tests and Cox proportional hazards analysis were used to assess 2-year MACE risk trajectory based on plasma protein levels. Results: The average participant age was 71 years (SD 10); 31.1% were female and 47.2% had diabetes. By the end of the two-year follow-up, 18.1% (*n* = 84) had experienced MACE. Of all proteins studied, only insulin-like growth factor-binding protein 1 (IGFBP-1) showed a significant elevation among patients who suffered MACE versus those who remained event-free (20.66 [SD 3.91] vs. 13.94 [SD 3.80] pg/mL; *p* = 0.012). IGFBP-1 remained a significant independent predictor of 2-year MACE occurrence in the multivariable Cox analysis (adjusted hazard ratio [HR] 1.57, 95% CI 1.21–1.97; *p* = 0.012). Subgroup analyses revealed that IGFBP-1 was significantly associated with 2-year MACE occurrence in both females (adjusted HR 1.52, 95% CI 1.16–1.97; *p* = 0.015) and males (adjusted HR 1.04, 95% CI 1.02–1.22; *p* = 0.045). Incorporating IGFBP-1 into the clinical risk prediction model significantly enhanced its predictive performance, with an increase in the AUROC from 0.73 (95% CI 0.71–0.75) to 0.79 (95% CI 0.77–0.81; *p* = 0.01), an NRI of 0.21 (95% CI 0.07–0.36; *p* = 0.014), and an IDI of 0.041 (95% CI 0.015–0.066; *p* = 0.008), highlighting the prognostic value of IGFBP-1. Kaplan–Meier analysis showed an increase in the cumulative incidence of 2-year MACE across IGFBP-1 tertiles. Patients in the highest IGFBP-1 tertile experienced a significantly higher event rate compared to those in the lowest tertile (log-rank *p* = 0.008). In the Cox proportional hazards analysis, the highest tertile of IGFBP-1 was associated with increased 2-year MACE risk compared to the lowest tertile (adjusted HR 1.81; 95% CI: 1.31–2.65; *p* = 0.001). Conclusions: Among the growth factors analyzed, IGFBP-1 emerged as the sole biomarker independently linked to the development of MACE over a two-year span in both female and male PAD patients. The addition of IGFBP-1 to clinical features significantly improved model predictive performance for 2-year MACE. Measuring IGFBP-1 levels may enhance risk stratification and guide the intensity of therapeutic interventions and referrals to cardiovascular specialists, ultimately supporting more personalized and effective management strategies for patients with PAD to reduce systemic vascular risk.

## 1. Introduction

Peripheral artery disease (PAD) impacts over 200 million people worldwide and involves atherosclerosis in the arteries supplying the lower extremities [1]. While PAD is often associated with limb complications, including amputation, cardiovascular events such as myocardial infarction and stroke remain the leading causes of death in this population [2]. This elevated risk stems from PAD’s strong association with coronary artery disease (CAD) and cerebrovascular disease (CVD), due to a shared foundation of systemic atherosclerosis [3]. Contributing factors include aging, hypertension, diabetes, high cholesterol, and tobacco use [4]. Consequently, early identification of PAD patients at elevated risk of major adverse cardiovascular events (MACE) is essential to guide timely referrals and implementation of strategies to reduce cardiovascular risk through multidisciplinary care [5]. A promising strategy for addressing this challenge involves the discovery of new biomarkers that can effectively predict cardiovascular events in the PAD population [6]. While our group has previously highlighted a number of biomarkers predictive of major adverse limb events (MALE) in PAD patients—including fatty acid-binding proteins [7], Cystatin C [8], and inflammatory proteins [9]—there remains a significant gap in research concerning markers that can reliably predict MACE in this high-risk group.

Growth factors are key signaling molecules that regulate fundamental biological processes such as cell proliferation, differentiation, and tissue regeneration [10]. Within the cardiovascular system, these proteins are instrumental in modulating pathways involved in angiogenesis, plaque formation, and cardiac remodeling, all of which are integral to both normal physiology and disease development [11]. Disruptions in growth factor signaling have been implicated in the onset and progression of cardiovascular conditions through their roles in metabolic imbalance, vascular dysfunction, abnormal vessel growth, and thrombosis [12,13,14]. Given their central involvement in these pathophysiological processes, circulating growth factors may serve as promising biomarkers for predicting MACE, particularly in individuals with PAD, where systemic atherosclerosis plays a dominant role [12,13,14]. One such protein, insulin-like growth factor-binding protein 1 (IGFBP-1), along with several others, has been linked to PAD, CAD, and CVD in prior research [15]. In fact, more than five growth factors have been consistently associated with these vascular conditions, supporting their relevance in cardiovascular risk assessment [14,15,16,17,18]. The five growth factors (IGFBP-1, tissue factor pathway inhibitor (TFPI), and bone morphogenetic protein (BMP)-4, -7, and -10) selected for this investigation were chosen based on a strong body of evidence connecting them to cardiovascular pathology [14,15,16,17,18]. Specifically, IGFBP-1, TFPI, and BMP-4, -7, and -10 have gained significant interest in recent years as potential biomarkers for cardiovascular disease given their demonstrated associations with systemic atherosclerosis, inflammation, thrombosis, endothelial dysfunction, and adverse cardiovascular events [14,15,16,17,18]. This specific panel of five proteins was selected over other biomarkers with established or emerging roles in PAD and MACE because of their strong mechanistic connections with cardiovascular pathophysiology, supporting their biological relevance in prognosticating systemic cardiovascular risk in PAD patients [14,15,16,17,18]. Although prior studies have linked these proteins to cardiovascular conditions, their specific predictive value for MACE in PAD patients remains underexplored [14,15,16,17,18]. This study differs from previous work on IGFBP-1 and cardiovascular disease because of the novelty of applying IGFBP-1 to MACE risk prediction in PAD patients specifically, which has not been undertaken previously [15]. Our group has previously shown that circulating proteins can effectively aid in diagnosing PAD and in forecasting MALE [9,19]. However, their potential utility in predicting broader cardiovascular outcomes in this patient population has not yet been examined [9,19]. This study was designed to evaluate a broad spectrum of growth factors to identify those with prognostic value for MACE in PAD, with the overarching goal of enhancing risk stratification and guiding more intensive treatment strategies aimed at mitigating systemic atherosclerosis and preventing future cardiovascular events in the PAD population.

## 2. Materials and Methods

### 2.1. Ethics

Ethical approval for this study was obtained from the Unity Health Toronto Research Ethics Board, affiliated with the University of Toronto, Canada (REB #16-375), on 8 February 2017. All participants gave written informed consent prior to enrollment, and the study was conducted in accordance with the ethical standards outlined in the Declaration of Helsinki [20].

### 2.2. Study Design

This investigation was designed as a prognostic study, with results presented according to the TRIPOD+AI statement [21].

### 2.3. Recruitment

Participants were prospectively enrolled from the ambulatory vascular clinics at St. Michael’s Hospital between January 2018 and August 2019. Eligibility was limited to patients diagnosed with peripheral artery disease (PAD), confirmed by a Toe–Brachial Index (TBI) under 0.7 or an Ankle–Brachial Index (ABI) below 0.9, along with absent or diminished pedal pulses [22]. Patients were excluded if they had experienced recent cardiovascular or limb-related complications, including acute coronary syndrome, acute limb ischemia, or elevated troponin levels within the past three months.

### 2.4. Baseline Characteristics

At baseline, the study gathered comprehensive demographic and clinical information, including age, sex, known cardiovascular risk factors, laboratory results, and medication use. Hypertension was characterized by a diastolic pressure of 80 mmHg or higher, a systolic blood pressure of 130 mmHg or greater, or the administration of antihypertensive drugs [23,24]. Dyslipidemia was defined by triglycerides exceeding 1.7 mmol/L, total cholesterol levels above 5.2 mmol/L, or treatment with lipid-lowering agents [23,24]. Diagnosis of diabetes was based on either a hemoglobin A1c value of 6.5% or higher or ongoing antidiabetic therapy [23,24]. Additionally, participants’ smoking status—encompassing both current and former smokers—was documented, along with any history of CAD, congestive heart failure (CHF), or stroke. All definitions of cardiovascular risk factors adhered to the standards established by the American College of Cardiology [23,24]. Bloodwork and medications captured included creatinine, estimated glomerular filtration rate (eGFR) calculated based on the 2021 Chronic Kidney Disease Epidemiology Collaboration (CKD-EPI) equation [25], hemoglobin A1c (HbA1c), fasting glucose, low-density lipoprotein (LDL), total cholesterol/high-density lipoprotein (HDL) ratio, non-HDL, acetylsalicylic acid (ASA), and angiotensin-converting enzyme inhibitor (ACE-I) or angiotensin II receptor blocker (ARB).

### 2.5. Quantification of Plasma Protein Concentrations

At the time of enrollment, venous blood was collected from participants and preserved at −80 °C until analysis. On the day of processing, samples underwent centrifugation to separate plasma from whole blood. Plasma concentrations of five targeted circulating growth factors were then measured in duplicate utilizing the LUMINEX multiplex assay (Bio-Techne, Minneapolis, MN, USA) [26]. The proteins analyzed—IGFBP-1, TFPI, and BMP-4, -7, and -10—were selected due to their established roles in pathways related to systemic atherosclerosis, metabolic dysregulation, and endothelial dysfunction, as well as their known associations with cardiovascular disease [14,15,16,17,18]. The purpose of evaluating multiple growth factors was to uncover potential new biomarkers relevant to PAD. Prior to performing the assays, the MagPix analyzer (Luminex Corp., Austin, TX, USA) [27] was calibrated using Fluidics Verification and Calibration Bead Kits (Luminex Corp., Austin, TX, USA) [28] to ensure optimal performance. To reduce variability between assays, all plasma samples were analyzed simultaneously on a single day. The coefficients of variation for both intra-assay and inter-assay measurements were kept under 10%. Data collection and analysis were performed using Luminex xPONENT software version 4.3, with a minimum of 50 beads captured per target protein [29].

### 2.6. Follow-Up and Outcomes

Participants attended routine follow-up visits at one and two years after their initial enrollment, with additional appointments scheduled as needed in response to emerging clinical concerns. The total number of clinic visits per patient during the 2-year follow-up period ranged from 2 to 6 based on clinical need. The main outcome measured in this study was the occurrence of MACE within a two-year follow-up period. This composite endpoint included myocardial infarction (MI), stroke, and death, with event verification conducted via direct clinical follow-up. MI was diagnosed based on a dynamic change in cardiac troponin levels—specifically, at least one value exceeding the 99th percentile upper reference limit—accompanied by at least one of the following clinical or diagnostic findings: (a) symptoms indicative of myocardial ischemia, (b) new ischemic alterations on electrocardiography, (c) development of pathological Q waves, (d) imaging evidence of new myocardial damage or regional wall motion abnormalities suggestive of ischemia, or (e) confirmation of coronary thrombus via angiography or autopsy [30]. This definition adheres to the international criteria set by the European Society of Cardiology, American College of Cardiology, American Heart Association, and World Heart Federation [30]. Stroke was defined as death of neural tissue in the brain, retina, or spinal cord due to ischemia, established by either (a) objective evidence from pathology, imaging, or other diagnostic modalities showing ischemic injury in a defined vascular territory, or (b) clinical symptoms consistent with focal ischemic damage that lasted 24 h or longer or led to death, with other causes ruled out [31]. This definition aligns with the standards provided by the American Heart Association and the American Stroke Association [31]. Mortality was captured as all-cause death. Over the 2 years of follow-up, patients received optimal guideline-directed treatment, ensuring that their blood pressure values were on target, blood sugars and dyslipidemia were under control, and PAD was being treated optimally by a vascular specialist with regular follow-up [32].

### 2.7. Statistical Analysis

Baseline demographic and clinical characteristics, as well as event rates, were summarized using means with standard deviations (SDs) for continuous variables and frequencies with percentages for categorical variables. Baseline characteristics were reported for the whole cohort and stratified by females and males. Differences in plasma protein concentrations between patients with and without MACE over the two-year follow-up were assessed using the Mann–Whitney U test. Proteins demonstrating significantly different expression levels between these groups were further evaluated for their prognostic value. Specifically, Cox proportional hazards regression was used to examine the association between each protein and 2-year MACE, adjusting for potential confounders including age, sex, hypertension, dyslipidemia, diabetes, smoking history (current and former), CHF, CAD, prior stroke, creatinine, eGFR, HbA1c, fasting glucose, LDL, total cholesterol/HDL ratio, non-HDL, ASA, and ACE-I/ARB. Plasma protein concentrations were treated as continuous variables in the predictive models, with hazard ratios (HRs) estimated per 1 pg/mL increment to evaluate their relationship with 2-year MACE. Recognizing that female patients with PAD often face a higher risk of adverse outcomes, subgroup analyses using Cox proportional hazards models were conducted to examine the prognostic value of circulating proteins specifically in women [33]. The purpose of this analysis was to identify biomarkers with potential prognostic significance for this high-risk and often understudied group. The prognostic value of adding the significant biomarker, IGFBP-1, to baseline characteristics was assessed using net reclassification improvement (NRI), which quantifies how well a new model correctly reclassifies subjects [34]. Specific to this study, NRI quantifies how much the addition of IGFBP-1 to clinical features improves model performance for predicting 2-year MACE [34]. The integrated discrimination improvement (IDI) was also calculated to assess the added value of IGFBP-1 in modeling performance when compared to using clinical features alone [35]. The primary model evaluation metric was area under the receiver operating characteristic curve (AUROC), a validated metric that considers both sensitivity and specificity [36]. AUROCs were compared using DeLong’s test [37]. The study population was then divided into three groups—low, medium, and high—according to tertiles of plasma IGFBP-1 concentrations, the sole protein showing a significant association with 2-year MACE. Kaplan–Meier survival analysis was performed to assess the time to MACE over two years, with group differences evaluated using the log-rank test and Cox proportional hazards models adjusted for baseline variables. This stratified approach was designed to further elucidate the prognostic implications of IGFBP-1, offering clinicians insight into how varying protein concentrations may influence patients’ risk trajectories for MACE. A two-sided *p*-value < 0.05 was considered statistically significant. All statistical analyses were performed using SPSS Statistics, version 23 (IBM Corp., Armonk, NY, USA) [38].

## 3. Results

### 3.1. Patients

**Figure 1:**
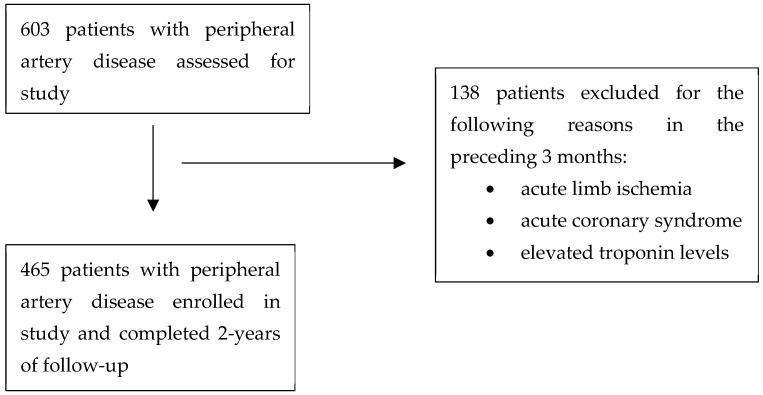
Flow diagram of patient selection.

A total of 465 patients with PAD were enrolled in the study (Figure 1). The mean age of the cohort was 71 years (SD 10), and 145 patients (31.1%) were female. As expected in a PAD population, cardiovascular comorbidities were highly prevalent: hypertension in 84.6% of patients, dyslipidemia in 82.3%, diabetes in 47.2%, past smoking history in 57.9%, and current smoking in 23.6%. Additionally, 4.7% had a history of CHF, 39.0% had CAD, and 19.7% had experienced a previous stroke. Baseline characteristics of the whole cohort, also stratified by females and males, are presented in Table 1. Given the relatively high rates of cardiovascular comorbidities in both females and males, aggressive risk-reduction strategies are critical in both groups to improve outcomes. In particular, the elevated mean creatinine of 98.61 (SD 69.96) µmol/L and reduced eGFR of 49.39 (SD 21.52) mL/min/1.73 m^2^ indicates decreased renal function in this PAD cohort, which was adjusted for in the multivariable analysis as this can influence IGFBP-1 levels.

### 3.2. Major Adverse Cardiovascular Events

Over the two-year follow-up, 84 participants (18.1%) experienced at least one MACE. Specifically, MI occurred in 70 individuals (15.0%), stroke was reported in 22 cases (4.7%), and 5 patients (1.2%) died from any cause during the study period (Table 2).

### 3.3. Plasma Concentrations of Growth Factors

Of the five growth factors evaluated, only IGFBP-1 was significantly elevated in patients with PAD who developed MACE within two years, compared to those who did not (20.66 [SD 3.91] vs. 13.94 [SD 3.80] pg/mL, *p* = 0.012). The remaining proteins showed no statistically significant differences between the two groups (Table 3).

### 3.4. Associations Between Growth Factors and Major Adverse Cardiovascular Events

After adjusting for baseline characteristics, IGFBP-1 plasma concentration remained independently associated with 2-year MACE in patients with PAD (adjusted HR 1.57; 95% CI 1.21–1.97; *p* = 0.012). None of the other circulating proteins demonstrated a statistically significant association with 2-year MACE (Table 4). In subgroup analyses by sex, IGFBP-1 remained significantly associated with 2-year MACE in both female patients (adjusted HR 1.52; 95% CI 1.16–1.97; *p* = 0.015) and male patients (adjusted HR 1.04; 95% CI 1.02–1.22; *p* = 0.045). The clinical importance of the modest HR for IGFBP-1 in the male subgroup is unclear and requires further investigation with larger cohorts. Sensitivity analysis excluding early MACE outcomes and stratifying by diabetes did not significantly change the results. The interaction between IGFBP-1 and patient sex was not statistically significant (*p* = 0.331). Similarly, interaction effects with patient sex for the remaining proteins were not statistically significant: BMP-4 (*p* = 0.483), BMP-7 (*p* = 0.120), BMP-10 (*p* = 0.743), and TFPI (*p* = 0.392).

The addition of IGFBP-1 to the baseline clinical model including all baseline characteristics improved discrimination for 2-year MACE. The AUROC (95% CI) increased from 0.73 (0.71–0.75) in the clinical model to 0.79 (0.77–0.81) in the model including IGFBP-1 (*p* = 0.01). The IDI was 0.041 (95% CI: 0.015–0.066; *p* = 0.008) and the NRI was 0.21 (95% CI: 0.07–0.36; *p* = 0.014) These findings demonstrate that incorporating IGFBP-1 alongside clinical variables significantly enhanced the predictive accuracy of the model for 2-year MACE, underscoring the biomarker’s prognostic importance.

### 3.5. Kaplan–Meier Analysis

**Figure 2:**
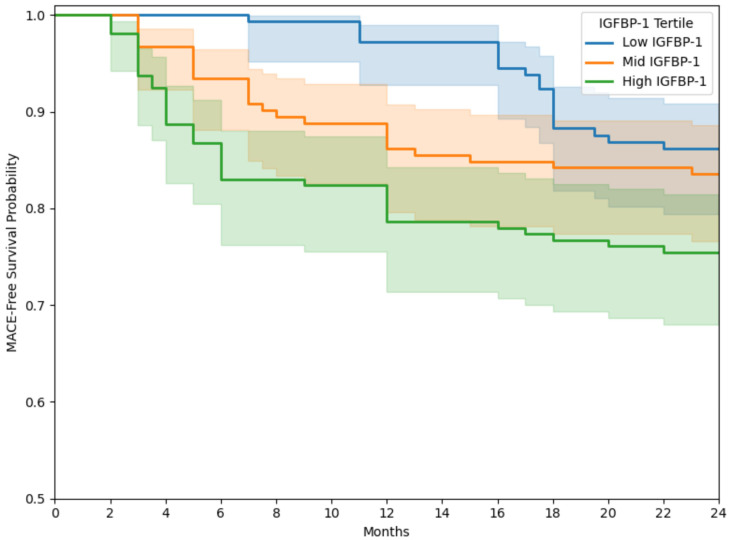
Kaplan–Meier curves for 2-year MACE stratified by IGFBP-1 tertiles in patients with PAD. Abbreviations: MACE (major adverse cardiovascular event); IGFBP-1 (insulin-like growth factor-binding protein 1); PAD (peripheral artery disease).

Kaplan–Meier analysis showed an increase in the cumulative incidence of 2-year MACE across IGFBP-1 tertiles. Patients in the highest IGFBP-1 tertile experienced a significantly higher event rate compared to those in the lowest tertile (log-rank *p* = 0.008) (Figure 2). In the Cox proportional hazards analysis, the highest tertile of IGFBP-1 was associated with increased risk of 2-year MACE compared to the lowest tertile (adjusted HR 1.81; 95% CI: 1.31–2.65; *p* = 0.001). On the other hand, the middle tertile was not significantly different from the lowest tertile (adjusted HR 1.22; 95% CI: 0.87–1.71; *p* = 0.241).

## 4. Discussion

### 4.1. Summary of Findings

This study identified IGFBP-1 as a circulating growth factor independently linked to the occurrence of MACE within two years in patients with PAD, suggesting its promise as a prognostic biomarker. There were several notable findings. First, out of the five growth factors evaluated, IGFBP-1 was uniquely elevated in patients who went on to experience MACE compared to those who remained event-free. Second, IGFBP-1 remained independently associated with 2-year MACE after adjusting for baseline demographic and clinical characteristics. Third, in subgroup analyses, IGFBP-1 was significantly associated with MACE in both female and male PAD patients. Given the higher risk of adverse events in female PAD patients and the limited exploration of sex-specific biomarkers in this population, this finding underscores the potential of IGFBP-1 as a particularly valuable prognostic marker in high-risk female patients [33]. Fourth, IGFBP-1 significantly improved model predictive performance for 2-year MACE compared to clinical features alone. Fifth, individuals with plasma IGFBP-1 concentrations in the top tertile demonstrated a greater incidence of MACE over two years compared to those in the bottom tertile. Collectively, our findings support the clinical relevance of IGFBP-1 for risk stratification in PAD, enabling the identification of individuals who may benefit from more intensive cardiovascular risk-reduction strategies to improve outcomes.

### 4.2. Comparison with the Existing Literature

Lewitt and colleagues (2024) reviewed the role of IGFBP-1 in cardiovascular disease, emphasizing its involvement in cellular growth, signaling, and metabolic regulation—pathways central to angiogenesis, atherosclerosis, and thrombosis [15]. Multiple studies have demonstrated associations between IGFBP-1 and various forms of cardiovascular disease. For instance, the Framingham Heart Study, which followed 3523 individuals over nearly three decades, found that elevated IGFBP-1 levels predicted cardiovascular mortality [39]. Similarly, Ritsinger et al. (2018) reported that increased IGFBP-1 levels after acute MI were associated with long-term all-cause mortality over 11.6 years of follow-up [40]. Wallander and colleagues also demonstrated that high IGFBP-1 levels were linked to increased cardiovascular morbidity and mortality in patients with type 2 diabetes following MI [41]. In patients with a first acute MI, elevated IGFBP-1 levels predicted subsequent heart failure development [42]. Within carotid arteries, IGFBP-1 has been demonstrated to be associated with intima–media thickness, which may predispose patients with carotid stenosis and stroke [43]. Aberg et al. (2023) found that higher serum IGFBP-1 levels were correlated with poorer long-term functional outcomes and increased mortality following stroke [44]. Genetic studies have further supported IGFBP-1’s role in cerebrovascular disease, with Yao et al. (2020) linking a single-nucleotide polymorphism in the IGFBP-1 gene to hemorrhagic stroke risk [45]. Histologically, IGFBP-1 has been detected in human carotid plaques, potentially contributing to plaque inflammation and instability [46]. Despite this growing body of evidence, the role of IGFBP-1 in PAD remains underexplored—a gap underscored in Lewitt et al.’s 2024 review [15]. Resanovic et al. reported that hyperbaric oxygen therapy increased IGFBP-1 levels in patients with type 1 diabetes and peripheral vascular complications [47]. Brevetti and colleagues showed that insulin-like growth factors (IGFs) were associated with the presence and severity of PAD through their modulation of plaque progression [48]. Our study builds on these findings by showing that IGFBP-1 is associated with increased 2-year MACE risk in patients with PAD, highlighting its potential role in the progression of atherosclerosis in multiple arterial beds. This is critically important given that the role of IGFBP-1, a well-established biomarker in CAD and CVD, remains understudied in PAD [15]. Furthermore, most patients enrolled in studies of IGFBP-1 related to cardiovascular disease are male [15]. By demonstrating that IGFBP-1 can act as a PAD biomarker in females, we have expanded our understanding of the potential role of IGFBP-1 in PAD care for females, who are known to be at higher risk of adverse events [33]. Our group recently showed that circulating proteins can be effective prognostic biomarkers for MALE risk in patients with PAD [6]. Extending these findings, the current study demonstrates that circulating IGFBP-1 is associated with broader systemic cardiovascular outcomes in the PAD population. These insights emphasize the importance of further mechanistic studies to explore the interplay between IGFBP-1 and the pathophysiology of PAD, CAD, and CVD. Ultimately, this could lead to the identification of novel therapeutic targets and refined strategies for personalized risk stratification and disease management in PAD.

### 4.3. Explanation of Findings

Insulin and related peptides, including IGFs, perform diverse physiological functions, particularly in metabolic regulation and cardiovascular homeostasis [49]. IGF-binding proteins (IGFBPs) bind IGFs with high affinity, modulating their bioavailability and activity [49]. These molecular interactions are essential in regulating both normal physiological functions and disease processes, such as metabolic disorders and cardiovascular conditions [49]. IGFBP-1, a 30 kDa protein predominantly synthesized by the liver, is one of six high-affinity IGFBPs [50]. It has conserved cysteine residues linked by disulfide bonds located in its N- and C-terminal regions, along with serine phosphorylation sites within the central domain [50]. The phosphorylation of IGFBP-1 increases its binding affinity for IGFs, thereby limiting IGF availability to type 1 IGF receptors, insulin receptors, and hybrid receptor complexes [50]. Circulating IGFBP-1 is primarily found in a phosphorylated form and, upon crossing the endothelium, inhibits IGF activity in peripheral tissues [50]. Under physiological conditions, plasma insulin levels are the primary regulators of IGFBP-1 concentration, with lower insulin levels typically associated with higher IGFBP-1 levels [50]. IGFBP-1 levels are also influenced by sex and age, with higher concentrations observed in females and older adults [15]. This is particularly relevant in the context of our findings given that we showed that IGFBP-1 was significantly associated with 2-year MACE in female PAD patients. Therefore, although physiologically elevated in females, IGFBP-1 may also play a role in the pathophysiology of systemic atherosclerosis in female PAD patients. Given that the mean age of females in the cohort was 73 years, the impact of menopausal changes associated with increased cardiovascular events is important and warrants further investigation in future studies assessing prognostic biomarkers for women with PAD [51]. IGFBP-1 may offer a valuable tool for addressing longstanding sex disparities in PAD care and outcomes [52]. Women with PAD are often undertreated due to atypical symptom presentation and are less likely to be referred for additional testing or interventions compared to men [52]. By providing an objective, biomarker-based method for risk assessment, IGFBP-1 could improve early risk stratification in female patients [52]. Furthermore, incorporating IGFBP-1 into clinical decision-making may facilitate more equitable treatment strategies, ultimately narrowing the gap in cardiovascular outcomes between women and men with PAD [52]. Beyond its role in IGF regulation, IGFBP-1 also exerts direct effects on metabolic processes and vascular function [53]. In both obese and non-obese mouse models, IGFBP-1 overexpression was associated with reduced blood pressure, improved insulin sensitivity, enhanced vasodilation, increased endothelial nitric oxide production, and protection against atherosclerosis [53]. Given that patients with PAD often have comorbid metabolic disorders such as diabetes, IGFBP-1 may serve as an integrated biomarker reflecting broader cardiovascular and metabolic health [53]. Genetic deletion of IGFBP-1 in mice impaired endothelial regeneration following vascular injury, highlighting its necessity for vascular repair [54]. Moreover, IGFBP-1 overexpression promoted limb perfusion and angiogenesis in insulin-resistant mice [54]. Overall, in vivo studies show that loss of IGFBP-1 plays a causal role in the development of cardiometabolic disorders, while increasing IGFBP-1 promotes neovascularization in response to ischemia [54]. The relationship between IGFBP-1 and MACE is complex, given studies suggesting that lower levels are associated with poor metabolic profiles and IGFBP-1 overexpression can be protective against atherosclerosis [53,54]. Our finding of elevated IGFBP-1 levels being associated with increased 2-year MACE risk can be reconciled with the existing literature as a compensatory mechanism for cardiovascular protection in high-risk patients. Specifically, we hypothesize that PAD patients with significant risk factors for systemic cardiovascular events may upregulate IGFBP-1 expression as a mechanism to support metabolic homeostasis, vascular repair, and endothelial function to reduce MACE risk. These mechanistic insights help to contextualize our findings, in which elevated IGFBP-1 levels were associated with increased 2-year MACE in PAD patients. By modulating vascular repair, endothelial function, and metabolic homeostasis, IGFBP-1 may play a critical role in the progression of systemic atherosclerosis. As such, IGFBP-1 represents a promising prognostic biomarker for MACE in patients with PAD and may serve as a potential therapeutic target in the future.

Other growth factors analyzed in this study included TFPI, BMP-4, BMP-10, and BMP-7. We did not find associations between these circulating proteins and 2-year MACE in patients with PAD. Although TFPI, a protein involved in regulating coagulation, has been hypothesized to be associated with cardiovascular disease, its predictive potential for adverse cardiovascular events is unclear [55]. Similarly, BMP-4, BMP-10, and BMP-7 have been implicated in cardiovascular physiology and pathology, including vascular inflammation, atherosclerosis, and calcification; however, their prognostic value for systemic cardiovascular risk in patients with PAD has not been established [16]. Our findings align with the existing literature and were not unexpected. The non-significant findings for these proteins may be attributed to study power, cohort characteristics, and disease contexts. Larger studies in non-PAD cohorts with CAD or CVD may provide further information regarding the prognostic value of these growth factors for systemic cardiovascular risk.

The assay developed in this study is currently for research use only. Translation to clinical practice requires additional validation studies assessing the prognostic value of IGFBP-1 in a prospective setting with larger patient cohorts. Moreover, it will be important to evaluate the incremental value of this biomarker over existing clinical risk stratification tools for systemic cardiovascular risk, such as the Revised Cardiac Risk Index [56]. We hypothesize that the addition of biologically relevant biomarkers to existing risk prediction tools that use only clinical features will improve predictive performance [57]. Integration of IGFBP-1 testing into routine clinical practice could be achieved by systematically addressing several key barriers, including technical, logistical, and cost-related challenges. On the technical front, standardized and validated assays must be widely available, with consistent calibration and quality control across laboratories to ensure reliable results. Logistically, incorporation into existing clinical workflows will require streamlined sample processing, timely result reporting, and appropriate training of clinical staff to interpret and act on findings. Cost-wise, affordability must be addressed through bulk reagent procurement, reimbursement strategies, and demonstration of clinical utility in reducing downstream healthcare expenditures. Addressing these elements collectively could facilitate broader adoption of IGFBP-1 testing and enable its use in disease monitoring, risk stratification, and clinical decision-making. This personalized risk assessment approach may improve clinical care and cardiovascular outcomes in patients with PAD.

### 4.4. Limitations

Several limitations should be acknowledged in this study. First, as it was performed at a single academic institution, the generalizability of the findings requires further validation in broader and more diverse populations across different healthcare environments. Second, the follow-up duration was limited to two years, capturing relevant short- to mid-term cardiovascular events; however, extended monitoring over 5 to 10 years or longer is needed to fully elucidate the long-term prognostic significance of IGFBP-1, particularly given the chronic and progressive nature of PAD, CVD, and CAD. Third, the sample size was insufficient to allow detailed analysis of the association of IGFBP-1 with individual MACE components. Larger studies with more events are needed to determine whether IGFBP-1 can predict MI, stroke, and death individually. Fourth, IGFBP-1 measurement is currently limited to research settings and is not yet widely available in routine clinical practice. Therefore, additional translational and implementation research is needed to evaluate the feasibility, cost-effectiveness, and clinical utility of incorporating IGFBP-1 testing into standard care pathways for patients with PAD.

## 5. Conclusions

Our study revealed that IGFBP-1 is a circulating growth factor independently associated with the occurrence of MACE over two years of follow-up among patients with PAD, highlighting its potential role as a biomarker for systemic atherosclerosis progression. Importantly, IGFBP-1 was significantly associated with MACE risk in both female and male PAD patients on subgroup analysis. This finding is particularly noteworthy given that female PAD patients have historically been understudied despite being at higher risk of adverse events. The identification of IGFBP-1 as a prognostic biomarker in this population may therefore represent an important step toward addressing sex-based disparities in PAD care. Additionally, IGFBP-1 significantly improved model predictive performance for 2-year MACE compared to clinical features alone. Overall, our findings support the clinical utility of IGFBP-1 for MACE risk stratification in PAD, which could enhance targeted cardiovascular risk-reduction strategies. High-risk individuals identified through IGFBP-1 screening could benefit from earlier and more aggressive intervention, including referral to cardiology, neurology, and vascular medicine specialists. This is particularly important as most patients with PAD ultimately die from MI or stroke. Lastly, our findings highlight the necessity for further research investigating the mechanistic involvement of IGFBP-1 in systemic atherosclerosis. Gaining deeper insights into these biological pathways could inform novel targeted treatments for PAD, CVD, and CAD.

## Figures and Tables

**Table 1:** Baseline characteristics of patients with peripheral artery disease.

	Whole PAD Cohort (*n* = 465)	Males (*n* = 320)	Females (*n* = 145)
Age, years, mean (SD)	71 (10)	69 (10)	73 (10)
Female sex	145 (31.1)	0 (0)	145 (100)
Hypertension	394 (84.6)	271 (84.7)	123 (84.8)
Dyslipidemia	383 (82.3)	269 (84.1)	114 (78.6)
Diabetes	220 (47.2)	164 (51.3)	56 (38.6)
Past smoking	269 (57.9)	186 (58.1)	83 (57.2)
Current smoking	110 (23.6)	92 (28.8)	18 (12.4)
Congestive heart failure	22 (4.7)	16 (5.0)	6 (4.1)
Coronary artery disease	181 (39.0)	150 (46.9)	31 (21.4)
Previous stroke	92 (19.7)	61 (19.1)	31 (21.4)
Creatinine, µmol/L, mean (SD)	98.61 (69.96)	98.07 (40.33)	100.08 (119.33)
eGFR, mL/min/1.73 m^2^, mean (SD)	49.39 (21.52)	49.37 (21.52)	49.26 (21.74)
HbA1c, %, mean (SD)	9.53 (25.95)	10.61 (30.61)	6.79 (1.67)
Fasting glucose, mmol/L, mean (SD)	8.23 (1.97)	8.48 (2.10)	7.13 (0.72)
LDL, mmol/L, mean (SD)	1.69 (0.7)	1.74 (0.78)	1.60 (0.52)
Total cholesterol/HDL ratio, mean (SD)	3.3 (1.53)	3.53 (1.74)	2.81 (0.79)
Non-HDL, mmol/L, mean (SD)	2.54 (0.97)	2.60 (1.08)	2.41 (0.67)
ASA	265 (57.0)	193 (60.3)	72 (49.7)
ACE-I/ARB	263 (56.6)	195 (60.9)	68 (46.9)

Data are shown as n (%) unless noted otherwise. Abbreviations: PAD, peripheral artery disease; SD, standard deviation; HbA1c, hemoglobin A1c; LDL, low-density lipoprotein; eGFR, estimated glomerular filtration rate; ASA, acetylsalicylic acid; ACE-I/ARB, angiotensin-converting enzyme inhibitor or angiotensin receptor blocker; HDL, high-density lipoprotein; Non-HDL, non-high-density lipoprotein.

**Table 2:** Major adverse cardiovascular events over 2 years in patients with peripheral artery disease.

	Patients with PAD (*n* = 465)
Major adverse cardiovascular event	84 (18.1)
Myocardial infarction	70 (15.0)
Stroke	22 (4.7)
Death	5 (1.2)

Values are reported as numbers (%). Abbreviations: SD (standard deviation); PAD (peripheral artery disease).

**Table 3:** Plasma protein concentrations in individuals with vs. without major adverse cardiovascular events.

	No MACE (*n* = 381)	MACE (*n* = 84)	*p*-Value
**IGFBP-1**	**13.94 (3.80)**	**20.66 (3.91)**	**0.012**
TFPI	20.58 (11.52)	21.20 (11.80)	0.062
BMP-4	10.98 (5.02)	11.5 (5.2)	0.087
BMP-10	125.91 (107.27)	130.5 (110.0)	0.089
BMP-7	95.8 (90.26)	98.4 (92.5)	0.105

Protein concentrations reported as mean (standard deviation), pg/mL. Bolded row is statistically significant (*p* < 0.05). Abbreviations: IGFBP-1 (insulin-like growth factor-binding protein 1); TFPI (tissue factor pathway inhibitor); BMP (bone morphogenetic protein); MACE (major adverse cardiovascular event).

**Table 4:** Associations between circulating growth factors and 2-year major adverse cardiovascular events.

	Adjusted Hazard Ratio	95% CI	*p*-Value
**IGFBP-1**	**1.59**	**(1.24–2.09)**	**0.007**
BMP-4	0.89	(0.75–1.06)	0.386
BMP-7	1.12	(0.98–1.45)	0.192
BMP-10	1.08	(0.80–1.31)	0.611
TFPI	0.92	(0.79–1.19)	0.420

Adjusted for all baseline characteristics. Hazard ratios were calculated for every 1 pg/mL increase in plasma protein concentration. Bolded row is statistically significant (*p* < 0.05). Abbreviations: IGFBP-1 (insulin-like growth factor-binding protein 1); TFPI (tissue factor pathway inhibitor); BMP (bone morphogenetic protein); CI (confidence interval).

## Data Availability

The original contributions presented in the study are included in the article; further inquiries can be directed to the corresponding author.

## References

[1] Olin J.W., Sealove B.A. (2010). Peripheral Artery Disease: Current Insight Into the Disease and Its Diagnosis and Management. Mayo Clin. Proc..

[2] Horváth L., Németh N., Fehér G., Kívés Z., Endrei D., Boncz I. (2022). Epidemiology of Peripheral Artery Disease: Narrative Review. Life.

[3] Grenon S.M., Vittinghoff E., Owens C.D., Conte M.S., Whooley M., Cohen B.E. (2013). Peripheral Artery Disease and Risk of Cardiovascular Events in Patients with Coronary Artery Disease: Insights from the Heart and Soul Study. Vasc. Med..

[4] Adhikary D., Barman S., Ranjan R., Stone H. (2022). A Systematic Review of Major Cardiovascular Risk Factors: A Growing Global Health Concern. Cureus.

[5] Jennings C., Astin F. (2017). A Multidisciplinary Approach to Prevention. Eur. J. Prev. Cardiol..

[6] Li B., Shaikh F., Zamzam A., Syed M.H., Abdin R., Qadura M. (2024). A Machine Learning Algorithm for Peripheral Artery Disease Prognosis Using Biomarker Data. iScience.

[7] Li B., Zamzam A., Syed M.H., Jahanpour N., Jain S., Abdin R., Qadura M. (2022). Urinary Fatty Acid Binding Protein 3 Has Prognostic Value in Peripheral Artery Disease. Front. Cardiovasc. Med..

[8] Li B., Zamzam A., Syed M.H., Jahanpour N., Jain S., Abdin R., Qadura M. (2022). Urinary Cystatin C Has Prognostic Value in Peripheral Artery Disease. Biomolecules.

[9] Li B., Djahanpour N., Zamzam A., Syed M.H., Jain S., Arfan S., Abdin R., Qadura M. (2022). The Prognostic Capability of Inflammatory Proteins in Predicting Peripheral Artery Disease Related Adverse Events. Front. Cardiovasc. Med..

[10] Stone W.L., Leavitt L., Varacallo M.A. (2025). Physiology, Growth Factor. StatPearls.

[11] Castro-Diehl C., Song R.J., Sawyer D.B., Wollert K.C., Mitchell G.F., Cheng S., Vasan R.S., Xanthakis V. (2021). Circulating Growth Factors and Cardiac Remodeling in the Community: The Framingham Heart Study. Int. J. Cardiol..

[12] Domouzoglou E.M., Naka K.K., Vlahos A.P., Papafaklis M.I., Michalis L.K., Tsatsoulis A., Maratos-Flier E. (2015). Fibroblast Growth Factors in Cardiovascular Disease: The Emerging Role of FGF21. Am. J. Physiol. Heart Circ. Physiol..

[13] Frangogiannis N.G. (2022). Transforming Growth Factor-β in Myocardial Disease. Nat. Rev. Cardiol..

[14] Florek K., Mendyka D., Gomułka K. (2024). Vascular Endothelial Growth Factor (VEGF) and Its Role in the Cardiovascular System. Biomedicines.

[15] Lewitt M.S., Boyd G.W. (2024). Insulin-like Growth Factor-Binding Protein-1 (IGFBP-1) as a Biomarker of Cardiovascular Disease. Biomolecules.

[16] Ye D., Liu Y., Pan H., Feng Y., Lu X., Gan L., Wan J., Ye J. (2023). Insights into Bone Morphogenetic Proteins in Cardiovascular Diseases. Front. Pharmacol..

[17] Perez-Shibayama C., Gil-Cruz C., Cadosch N., Lütge M., Cheng H.-W., De Martin A., Frischmann K., Joachimbauer A., Onder L., Papadopoulou I. (2024). Bone Morphogenic Protein-4 Availability in the Cardiac Microenvironment Controls Inflammation and Fibrosis in Autoimmune Myocarditis. Nat. Cardiovasc. Res..

[18] Ceelen D.C.H., Bracun V., van Essen B.J., Voors A.A., de Boer R.A., ter Maaten J.M., Masson S., Kastner P., Lang C.C., Suthahar N. (2025). Circulating Bone Morphogenetic Protein 10 as a Novel Marker of Atrial Stress and Remodelling in Heart Failure. Heart.

[19] Li B., Nassereldine R., Shaikh F., Younes H., AbuHalimeh B., Zamzam A., Abdin R., Qadura M. (2024). Inflammatory Protein Panel: Exploring Diagnostic Insights for Peripheral Artery Disease Diagnosis in a Cross-Sectional Study. Diagnostics.

[20] World Medical Association (2013). World Medical Association Declaration of Helsinki: Ethical Principles for Medical Research Involving Human Subjects. JAMA.

[21] Collins G.S., Moons K.G.M., Dhiman P., Riley R.D., Beam A.L., Calster B.V., Ghassemi M., Liu X., Reitsma J.B., van Smeden M. (2024). TRIPOD+AI Statement: Updated Guidance for Reporting Clinical Prediction Models That Use Regression or Machine Learning Methods. BMJ.

[22] Gul F., Janzer S.F. (2021). Peripheral Vascular Disease. StatPearls.

[23] Grundy S.M., Stone N.J., Bailey A.L., Beam C., Birtcher K.K., Blumenthal R.S., Braun L.T., de Ferranti S., Faiella-Tommasino J., Forman D.E. (2019). 2018 AHA/ACC/AACVPR/AAPA/ABC/ACPM/ADA/AGS/APhA/ASPC/NLA/PCNA Guideline on the Management of Blood Cholesterol. J. Am. Coll. Cardiol..

[24] Whelton P.K., Carey R.M., Aronow W.S., Casey D.E., Collins K.J., Dennison H.C., DePalma S.M., Gidding S., Jamerson K.A., Jones D.W. (2018). 2017 ACC/AHA/AAPA/ABC/ACPM/AGS/APhA/ASH/ASPC/NMA/PCNA Guideline for the Prevention, Detection, Evaluation, and Management of High Blood Pressure in Adults. J. Am. Coll. Cardiol..

[25] Delgado C., Baweja M., Crews D.C., Eneanya N.D., Gadegbeku C.A., Inker L.A., Mendu M.L., Miller W.G., Moxey-Mims M.M., Roberts G.V. (2022). A Unifying Approach for GFR Estimation: Recommendations of the NKF-ASN Task Force on Reassessing the Inclusion of Race in Diagnosing Kidney Disease. Am. J. Kidney Dis..

[26] Luminex Assays, Multiplex Immunoassays. https://www.bio-techne.com/.

[27] MAGPIX^®^ System | xMAP Instrument | Luminex Corporation. https://www.luminexcorp.com/magpix-system/.

[28] Luminex Assays—CA. www.thermofisher.com/ca/en/home/life-science/antibodies/immunoassays/procartaplex-assays-luminex.html.

[29] (2025). xPONENT^®^ Software for xMAP^®^ Instruments.

[30] Thygesen K., Alpert J.S., Jaffe A.S., Chaitman B.R., Bax J.J., Morrow D.A., White H.D. (2018). The Executive Group on behalf of the Joint European Society of Cardiology (ESC)/American College of Cardiology (ACC)/American Heart Association (AHA)/World Heart Federation (WHF) Task Force for the Universal Definition of Myocardial Infarction Fourth Universal Definition of Myocardial Infarction (2018). Circulation.

[31] Sacco R.L., Kasner S.E., Broderick J.P., Caplan L.R., Connors J.J., Culebras A., Elkind M.S.V., George M.G., Hamdan A.D., Higashida R.T. (2013). An Updated Definition of Stroke for the 21st Century: A Statement for Healthcare Professionals from the American Heart Association/American Stroke Association. Stroke.

[32] Gornik H.L., Aronow H.D., Goodney P.P., Arya S., Brewster L.P., Byrd L., Chandra V., Drachman D.E., Eaves J.M., Ehrman J.K. (2024). 2024 ACC/AHA/AACVPR/APMA/ABC/SCAI/SVM/SVN/SVS/SIR/VESS Guideline for the Management of Lower Extremity Peripheral Artery Disease: A Report of the American College of Cardiology/American Heart Association Joint Committee on Clinical Practice Guidelines. Circulation.

[33] Jelani Q.-U.-A., Petrov M., Martinez S.C., Holmvang L., Al-Shaibi K., Alasnag M. (2018). Peripheral Arterial Disease in Women: An Overview of Risk Factor Profile, Clinical Features, and Outcomes. Curr. Atheroscler. Rep..

[34] Kerr K.F., Wang Z., Janes H., McClelland R.L., Psaty B.M., Pepe M.S. (2014). Net Reclassification Indices for Evaluating Risk-Prediction Instruments: A Critical Review. Epidemiology.

[35] Pencina M.J., Demler O.V. (2012). Novel Metrics for Evaluating Improvement in Discrimination: Net Reclassification and Integrated Discrimination Improvement for Normal Variables and Nested Models. Stat. Med..

[36] Hajian-Tilaki K. (2013). Receiver Operating Characteristic (ROC) Curve Analysis for Medical Diagnostic Test Evaluation. Casp. J. Intern. Med..

[37] DeLong E.R., DeLong D.M., Clarke-Pearson D.L. (1988). Comparing the Areas under Two or More Correlated Receiver Operating Characteristic Curves: A Nonparametric Approach. Biometrics.

[38] (2021). SPSS Software.

[39] Ho J.E., Lyass A., Courchesne P., Chen G., Liu C., Yin X., Hwang S.-J., Massaro J.M., Larson M.G., Levy D. (2018). Protein Biomarkers of Cardiovascular Disease and Mortality in the Community. J. Am. Heart Assoc..

[40] Ritsinger V., Brismar K., Mellbin L., Näsman P., Rydén L., Söderberg S., Norhammar A. (2018). Elevated Levels of Insulin-like Growth Factor-Binding Protein 1 Predict Outcome after Acute Myocardial Infarction: A Long-Term Follow-up of the Glucose Tolerance in Patients with Acute Myocardial Infarction (GAMI) Cohort. Diab. Vasc. Dis. Res..

[41] Wallander M., Norhammar A., Malmberg K., Ohrvik J., Rydén L., Brismar K. (2007). IGF Binding Protein 1 Predicts Cardiovascular Morbidity and Mortality in Patients with Acute Myocardial Infarction and Type 2 Diabetes. Diabetes Care.

[42] Janszky I., Hallqvist J., Ljung R., Hammar N. (2010). Insulin-like Growth Factor Binding Protein-1 Is a Long-Term Predictor of Heart Failure in Survivors of a First Acute Myocardial Infarction and Population Controls. Int. J. Cardiol..

[43] Boquist S., Ruotolo G., Skoglund-Andersson C., Tang R., Björkegren J., Bond M.G., De Faire U., Brismar K., Hamsten A. (2008). Correlation of Serum IGF-I and IGFBP-1 and -3 to Cardiovascular Risk Indicators and Early Carotid Atherosclerosis in Healthy Middle-Aged Men. Clin. Endocrinol..

[44] Åberg D., Gadd G., Jood K., Redfors P., Stanne T.M., Isgaard J., Blennow K., Zetterberg H., Jern C., Åberg N.D. (2023). Serum IGFBP-1 Concentration as a Predictor of Outcome after Ischemic Stroke-A Prospective Observational Study. Int. J. Mol. Sci..

[45] Yao Y., Zhu H., Zhu L., Fang Z., Fan Y., Liu C., Tian Y., Chen Y., Tang W., Ren Z. (2020). A Comprehensive Contribution of Genetic Variations of the Insulin-like Growth Factor 1 Signalling Pathway to Stroke Susceptibility. Atherosclerosis.

[46] Wang J., Razuvaev A., Folkersen L., Hedin E., Roy J., Brismar K., Hedin U. (2012). The Expression of IGFs and IGF Binding Proteins in Human Carotid Atherosclerosis, and the Possible Role of IGF Binding Protein-1 in the Regulation of Smooth Muscle Cell Proliferation. Atherosclerosis.

[47] Resanović I., Gluvić Z., Zarić B., Sudar-Milovanović E., Vučić V., Arsić A., Nedić O., Šunderić M., Gligorijević N., Milačić D. (2020). Effect of Hyperbaric Oxygen Therapy on Fatty Acid Composition and Insulin-like Growth Factor Binding Protein 1 in Adult Type 1 Diabetes Mellitus Patients: A Pilot Study. Can. J. Diabetes.

[48] Brevetti G., Colao A., Schiano V., Pivonello R., Laurenzano E., Di Somma C., Lombardi G., Chiariello M. (2008). IGF System and Peripheral Arterial Disease: Relationship with Disease Severity and Inflammatory Status of the Affected Limb. Clin. Endocrinol..

[49] LeRoith D., Holly J.M.P., Forbes B.E. (2021). Insulin-like Growth Factors: Ligands, Binding Proteins, and Receptors. Mol. Metab..

[50] Forbes B.E., McCarthy P., Norton R.S. (2012). Insulin-Like Growth Factor Binding Proteins: A Structural Perspective. Front. Endocrinol..

[51] Anagnostis P., Mikhailidis D.P., Blinc A., Jensterle M., Ježovnik M.K., Schernthaner G.-H., Antignani P.L., Studen K.B., Šabović M., Poredos P. (2023). The Effect of Menopause and Menopausal Hormone Therapy on the Risk of Peripheral Artery Disease. Curr. Vasc. Pharmacol..

[52] Pabon M., Cheng S., Altin S.E., Sethi S.S., Nelson M.D., Moreau K.L., Hamburg N., Hess C.N. (2022). Sex Differences in Peripheral Artery Disease. Circ. Res..

[53] Rajwani A., Ezzat V., Smith J., Yuldasheva N.Y., Duncan E.R., Gage M., Cubbon R.M., Kahn M.B., Imrie H., Abbas A. (2012). Increasing Circulating IGFBP1 Levels Improves Insulin Sensitivity, Promotes Nitric Oxide Production, Lowers Blood Pressure, and Protects against Atherosclerosis. Diabetes.

[54] Haywood N.J., Slater T.A., Drozd M., Warmke N., Matthews C., Cordell P.A., Smith J., Rainford J., Cheema H., Maher C. (2020). IGFBP-1 in Cardiometabolic Pathophysiology—Insights from Loss-of-Function and Gain-of-Function Studies in Male Mice. J. Endocr. Soc..

[55] Brook R., Suleiman M., Rigano J., Lui B., Nandurkar H., Ho P., Lim H.Y. (2025). Tissue Factor Pathway Inhibitor Levels and Atherothrombotic Events in Patients with Chronic Kidney Disease or Diabetes. J. Thromb. Thrombolysis.

[56] Lee T.H., Marcantonio E.R., Mangione C.M., Thomas E.J., Polanczyk C.A., Cook E.F., Sugarbaker D.J., Donaldson M.C., Poss R., Ho K.K. (1999). Derivation and Prospective Validation of a Simple Index for Prediction of Cardiac Risk of Major Noncardiac Surgery. Circulation.

[57] Taylor J.M.G., Ankerst D.P., Andridge R.R. (2008). Validation of Biomarker-Based Risk Prediction Models. Clin. Cancer Res..

